# Parvovirus B19-induced severe anemia in heart transplant recipient: A case report

**DOI:** 10.1097/MD.0000000000028387

**Published:** 2021-12-23

**Authors:** Bijun Xu, Weimin Zhang, Ximing Qian, Shiqiang Wang, Fan He

**Affiliations:** Department of Cardiovascular Surgery, Sir Run Run Shaw Hospital, Zhejiang University, China.

**Keywords:** anemia, heart transplant, parvovirus B19

## Abstract

**Rationale:**

Human parvovirus B19 (B19V) is a non-enveloped single-stranded DNA virus associated with a variety of human diseases. Reports of B19V infection after cardiac transplantation are relatively rare.

**Patient concerns:**

We report a case of a 48-year-old women who underwent orthotopic heart transplant for dilated cardiomyopathy. She developed an anemia after cardiac transplantation. Anemia was most severe 2 months after surgery, with a decrease in reticulocyte count. Serological DNA test for parvovirus B19V was performed and the result was positive.

**Diagnoses:**

B19V infection.

**Interventions and outcomes:**

Intravenous immunoglobulin administration resulted in a resolution of the anemia. The patient's blood test results showed a normal hemoglobin and reticulocyte count 1 year after surgery.

**Lessons:**

Patients with parvovirus B19V infection may develop severe anemia after heart transplantation. The diagnosis mainly relies on viral DNA detection. Intravenous immunoglobulin is an effective treatment for viral infection.

## 1 Introduction

Human parvovirus B19 (B19V) is a non-enveloped single-stranded DNA virus associated with a variety of human diseases.^[1]^ B19V infection usually occurs in childhood, with 50% of adolescents developing antibodies to the virus before age 15.^[2]^ It has been reported that recipients are particularly susceptible to B19V infection after transplant operation, especially kidney and liver transplantation.^[3]^ However, reports of B19V infection after cardiac transplantation are relatively rare. Here, we report a patient with severe anemia caused by B19V infection after cardiac transplantation.

## 2 Case report

A 48-year-old female patient was admitted to our hospital 2 months ago because of dilated cardiomyopathy and heart transplantation operation was performed successfully. A triple anti-rejection regimen of tacrolimus, motimecocurphon, and prednisone was administered postoperatively. The patient began to show progressive decline in hemoglobin, which was 97 g/L on the first day after surgery, and reduced to 63 g/L 2 weeks later. Hemoglobin increased to 76 g/L after infusion of red blood cell suspension. Factors related to bleeding were excluded, and the patient was discharged 1 month after surgery.

During follow-up, the patient complained of intermittent palpitation accompanied by fatigue, without symptoms such as hematemesis, hematochezia, black feces, and weight loss. The result of hemoglobin was 59 g/L, and the patient was readmitted to hospital for further evaluation. Physical examination showed a pale face. Laboratory examination suggested a hemoglobin of 50 g/L, a hematocrit of 17.6% and a reticulocyte count of 0.23%. Serum iron ion value was 21.6 μmol/L, total iron binding capacity 43.10 μmol/L, transferrin saturation 50.1% and ferritin 506 μg/L. B19V infection was suspected and serological DNA test for B19V was performed. At the same time, the patient was given 2 units of red cell suspension but with no significant improvement in hemoglobin. Serological DNA results of B19V at 5 days suggested greater than 1 ×× 10^8^ copies/mL. B19V infection was confirmed. Treatment was started with intravenous immunoglobulin (IVIG) given at 300 mg/kg/d for 9 days. The symptoms of palpitations and fatigue began to improve and the hemoglobin was also increased. It increased to 117 g/L 1 month after re-admission. One year after the operation, the patient had a significant improvement in their quality of life, without chest tightness, shortness of breath, palpitation, dizziness, and other symptoms. Laboratory examination showed that the hemoglobin was 130 g/L, and the mean hemoglobin volume, hematocrit, mean erythrocyte volume, and reticulocyte count were all within the normal range.

## 3 Discussion

The etiology of post-heart transplant anemia is multifactorial and includes medications (e.g., those used as immunosuppressive and antimicrobial prophylaxis), perioperative bleeding, decrease in intestinal absorption of vitamins, renal failure, and low levels of erythropoietin, and elevated levels of hepcidin associated with inflammation and reduced availability of iron.^[4]^ However, the incidence of B19V infection after transplantation is not clear. Some studies have reported that it is about 0% to 58%,^[5–7]^ which occurs mostly after kidney transplantation. Studies have shown that among transplant recipients, 98.8% of patients infected with B19V have anemia, and some recipients may also develop fever, joint pain, and rash.^[8]^ However, anemia associated with B19V infection can be easily overlooked because the epidemiological risk factors are not obvious. Replication of the virus in erythroid progenitor cells induces lysis of infected cells and down-regulation of erythropoietin receptor expression. In immunosuppressive receptors, persistent lysis of erythrocyte precursors and reduced erythropoiesis may lead to severe chronic disorder of pure erythropoiesis. Therefore, transplant recipients with erythropoietin resistant anemia with decreased reticulocyte count should be suspected of B19V infection.^[9]^ Of course, clinicians should consider the possibility of B19V infection in patients with progressive hemoglobin decline and poor response to routine correction of anemia. Our case presented severe anemia after heart transplantation, but there was no evidence of blood loss, hemolysis, iron absorption disorder, and renal insufficiency. Moreover, the anemia symptom had no significant improvement after receiving 2 blood transfusions. After eliminating the above possible causes, a reticulocyte examination was performed and the result was significantly reduced. We suspected that the patient's anemia was caused by acute B19V infection, so serological DNA tests for B19V was performed and the results confirmed our suspicion.

There is currently no specific method for isolation of viruses from clinical specimens. Therefore, the diagnosis of the disease mainly relies on the detection of IgM and IgG antibodies and PCR detection of viral DNA.^[10]^ In immunocompetent individuals, diagnosis depends on serological detection of parvovirus B19V IgG and IgM. However, antibody responses are often absent in immunocompromised patients. Therefore, the diagnosis of B19V infection requires quantitative PCR detection of B19V DNA in peripheral blood, bone marrow samples, or biopsy tissue specimens.^[10]^ During the initial period of infection, B19V DNA is detectable at a high titer (>10^9^IU/mL) for 2 to 4 days, then dropped to between 10^2^ and 10^4^ IU/mL and usually disappeared by day 14.

IVIG has a good effect in the treatment of anemia induced by B19V infection after transplantation. Because immunoglobulin contains antibodies that neutralize B19V.^[11]^ A small number of patients who were not treated with IVIG but were given erythropoietin, iron supplements and blood transfusions also had better recovery of anemia.^[10]^ However, there is no consensus on the optimal dosing schedule and duration of IVIG treatment. Most of the patients received 300 ∼ 400 mg/kg/d IVIG for 7 ∼ 10 d, and some patients needed to extend the treatment time. Limited data suggest that a 2-day course of IVIG may be as effective as a 7-day course of 400 mg/kg/d as long as the total dose is at least 2 g/kg.^[12]^ Tacrolimus has a stronger immunosuppressive effect than cyclosporine A, the use of immunosuppressant should be minimized or the conversion of tacrolimus to cyclosporine A should be helpful after diagnosis of parvovirus B19V infection. However, Crabol et al^[12]^ believed that IVIG is an effective method for the treatment of B19V infection and may avoid the need to discontinue immunosuppression with tacrolimus. Our patient received a 9-day course of IVIG (300 mg/kg/d) without discontinuing tacrolimus, and his anemia also improved significantly. Of course, for patients with severe immunosuppression, IVIG combined with reduction or substitution of tacrolimus for cyclosporine A may lead to a faster recovery time.

## 4 Conclusion

Patients with parvovirus B19V infection may develop severe anemia after heart transplantation. The diagnosis mainly relies on viral DNA detection. IVIG is an effective treatment for viral infection. For patients with severe immunosuppression, the treatment regimen of immunosuppression should be adjusted simultaneously.

## Author contributions

**Conceptualization:** Ximing Qian.

**Methodology:** Shiqiang Wang.

**Supervision:** Wei-Min Zhang.

**Writing – original draft:** Bijun Xu.

**Writing – review & editing:** Fan He.
